# Editorial: Reviews in ethnopharmacology: 2023

**DOI:** 10.3389/fphar.2025.1589249

**Published:** 2025-04-01

**Authors:** Rajeev K. Singla, Irina Ielciu, Daniela Hanganu, Michel Frederich

**Affiliations:** ^1^ Department of Critical Care Medicine and Institutes for Systems Genetics, Frontiers Science Center for Disease-related Molecular Network, West China Hospital, Sichuan University, Chengdu, China; ^2^ School of Pharmaceutical Sciences, Lovely Professional University, Phagwara, Punjab, India; ^3^ Department of Pharmaceutical Botany, Faculty of Pharmacy, “Iuliu Haţieganu” University of Medicine and Pharmacy, Cluj-Napoca, Romania; ^4^ Department of Pharmacognosy, Faculty of Pharmacy, “Iuliu Haţieganu” University of Medicine and Pharmacy, Cluj-Napoca, Romania; ^5^ Pharmacognosy Laboratory, Center of Interdisciplinary Research on Medicine (CIRM), University of Liège, Liège, Belgium

**Keywords:** natural products, medicinal plants, bioactive agents, traditional medicine, ethnomedicine

Since centuries, natural products have long been used for the treatment and management of various diseases and disorders (Dubey and Singla, 2019; Singla et al., 2019a; Singla et al., 2023b). Natural products have been the goldmine of bioactive compounds that hold great significance clinically (Singla et al., 2019b; Wang et al., 2024). Considering the strong role of natural products in the field of drug discover, we have proposed the Research Topic “Reviews in Ethnopharmacology: 2023” in order to collate the literature in the field of ethnopharmacology. We have received a total of 65 manuscripts, out of which, 30 manuscripts have been successfully published in our Research Topic. A total of 232 authors have contributed in this Research Topic. This Research Topic has garnered significant attention from the readers and viewers, with 83K topic views, 56K article views, and 24K article downloads, as on 5th March, 2025.

The study of Wang et al. proposed the combination therapy of *Rehmannia glutinosa* DC. and *Lilium lancifolium* Thunb. for the treatment and management of depression. This combination is also associated with the traditional Chinese medicine, Baihe Dihuang Decoction. Bioactive compounds from this combination reported to inhibit serotonin or 5-hydroxytryptamine (5-HT) reuptake in various nerve endings and cell bodies, along with modulation of brain-derived neurotrophic factor (BDNF),Tyrosine kinase receptor B (TrkB), Neurotrophin P75 receptor (P75NRT), Phospholipase Cγ (PLCγ), Phosphatidylinositol 3 kinase (PI3K), Raf kinase (Raf), and other targeted genes for depression as described in this article. Orozco-Barocio et al. focused on the cancer prevention and treatment using phytochemicals derived from medicinal plants belonging to *Cactaceae* family. *Opuntia* genera is the most significant genera of this family. This article tends to give an overview on various genera of *Cactaceae* family and their therapeutic potential especially in relation to cancer. Rahman et al. focused on the bioactive metabolites derived from soybean and discussed their medicinal and pharmaceutical properties in their study. Among the valuable metabolites they have highlighted were genistein, phytic acid, dietary fibers, conjugated linoleic acid, pinitol, and others. Soybean-derived compounds have broad-spectrum therapeutic properties, including but not limited to anticancer, antidiabetic, antiviral, anti-bacterial, antihypertensive, anti-obesity. Kuang et al. focused on *Polygalae Radix* and discussed its botany, metabolites, pharmacological properties, toxicity, and other applications, reporting that more than 320 compounds have been isolated and characterized from this source and saponins, xanthones, and oligosaccharide esters were the major fraction out of all. Apart from the major role in neurodegenerative disorders, *Polygalae Radix* and its metabolites also possess the functionality as anti-oxidant, anti-cancer, anti-inflammatory, along with others. Chen et al. demonstrated that the extract from *Cynomorium songaricum* Rupr. which was enriched with flavonoids, has the capability to improve the physiological conditions occurred due to the cyclophosphamide-induced reproductive function damage in mice, and bisphenol A-induced TM3 cell damage. They have indicated that the mechanism of action is regulation of testosterone synthesis pathway. Li et al. focused on alkaloids derived from *Chelidonium majus* L. (regionally commonly known as bai qu cai, tuhuanglian (土黄连)). They provided information about 94 alkaloids in this manuscript and discussed their therapeutic potential, including but not limited to anti-microbial, anti-viral, anti-tumor, and others. Sun et al. focuses on the polysaccharides derived from *Eucommia ulmoides* and discussed their extraction, purification, structural characterization, and therapeutic applications. Over the years, patents associated with the *E. ulmoides*-derived polysaccharides have been receiving gradually increasing interest, indicating its market potential. Liu et al. focused on *Phyllanthus urinaria* L., its metabolites, and their therapeutic potential for the treatment and management of liver-associated diseases, including viral hepatitis and liver fibrosis. Authors have discussed various metabolites of *P. urinaria* L. like flavonoids, lignans, tannins, etc., and thoroughly discussed the mechanism of action associated with their therapeutic potential. Cai et al. demonstrated in a retrospective cohort study that the combinatorial effect of Fu Zheng Jie Du formula (FZJDF) combined with prone ventilation in the patients suffering from severe pneumonia. Adjuvant effect of FZJDF with prone ventilation has been well-documented through this study with the significant improvement in oxygenation. Qian et al. studied syringin, which is a phenylpropanoid glycoside, and widely occurring in various plants (23 families, 60 genera, 100 plant species), including *Acanthopanax senticosus* (Rupr. et Maxim.) Harms. It has also been a key component in various Chinese herbal medicines, including but not limited to Aidi injection, compound cantharidin capsule, and Tanreqing injection, which are being used clinically. It has broad-spectrum of therapeutic properties. Sadek et al. focused on the bee venom and elaborated as how it is a potential source with therapeutic and regenerative medical applications. This is a comprehensive review which discusses throughly about the bee venom, its metabolites, therapeutic potential, its related safety concerns, and nanoformulations for the mitigation of adverse effects and improving its clinical potential. Fu et al. highlighted the role of the traditional Chinese medicine-based extracts in the treatment and management of sepsis. This review article comprehensively covered the mechanisms associated with the anti-sepsis properties of traditional Chinese medicine. Qingwenbaidu decoction, Dachengqi decoction, and Xuebijing injection are among the traditional Chinese medicine being discussed in this manuscript. Ain et al. focused on neglected tropical disease, malaria and how the natural remedies can be used to treat and manage this protozoal disease. Although most of the clinically used medicine for malaria are either natural products or derived from natural products, but there seems to have emerging resistance. This encourages researchers to explore more natural resources like *Helianthus annuus* L., and compounds like cryptolepine and isoliquiritigenin to combat drug resistance issues. Zhao et al. performed a systematic review and metanalysis of Zao Ren An Shen prescription, a commercially utilized Chinese polyherbal formulation for the treatment and management of primary insomnia. Authors have adopted a new meta-analysis methodology, trial sequential analysis (TSA) for analysing the data. Although there is promising efficacy of this Zao Ren An Shen prescription in primary insomnia, the quality of evidence was limited. Thus, authors have recommended clinical researchers to conduct rigorously-designed randomized control trials, so as to validate and get quality evident data. Gao et al. studied the natural products which elicits protective effect against rheumatoid arthritis, primarily through their inhibitory effect on synovial angiogenesis. They have further discussed various pathways associated with synovial angiogenesis that have been impacted by natural products, including but not limited to *Tripterygium wilfordii* Hook. f.

Yu et al. focused on Traditional Chinese Medicine affecting age-related macular degeneration, with primary focus on mitophagy modulators. Various linked pathways like phosphatase and tensin homolog-induced kinase 1/Parkin, and others have also been discussed. Ma et al. performed a comprehensive review on Chinese botanical drugs which specifically target mitophagy for the treatment and management of diabetic kidney disease. Various linked pathways like ubiquitination-dependent mitophagy pathway, receptor-mediated mitophagy pathways, and membrane lipid-mediated signaling pathways have also been comprehensively discussed. Some of the Chinese botanical drugs discussed in that manuscript include Tangshen formula, Huangkui capsule, Esculetin, along with many others. Zhang et al. discussed various metabolites like nicotine, solanesol and cembranoid diterpenes, which were isolated from *Nicotiana tabacum* L. (tobacco). Such metabolites have been experimentally validated to potentially alleviate neurological disorders, inflammatory disorders, metabolic disorders, as explained in this article. The article covered the English and Chinese literature, spanning over 2 decades. Mottaghipisheh et al. focused on *Vitex trifolia* L. and discussed its ethnomedicinal values, phytochemistry, and therapeutic potential. Out of all the discussed metabolites, metabolites from terpenoids and flavonoids classes so far the most characterized from this plant. *Vitex trifolia* L. holds great potential and broad spectrum of pharmacological properties, including but not limited to hepatoprotective, anticancer and antiviral properties. Li et al. comprehensively discussed traditional Tibetan medicines and their therapeutic potential in treating and managing various lung diseases. In this review articles, they have compiled information about 586 Tibetan medicines, out of which, 33 have been experimentally studied for their effectiveness in lung diseases. Rhodiola, gentian, sea buckthorn, and liexiang dujuan, are some of the most important Tibetan medicines in treating lung diseases. Cui et al. performed Bayesian network meta-analysis and systematic review analysis to understand the efficacy of the combination therapy of Chinese patent medicine with calcium channel blockers for the treatment and management of essential hypertension. Some of the Chinese patent medicine discussed in this work are Tianma Gouteng Granule, Songling Xuemaikang Capsule, Qinggan Jiangya Capsule, along with others. Wang et al. prepared a systematic review article and also performed meta-analysis to understand the effectiveness of the protective potential of ligustrazine while treating ischemic stroke. Based on the 32 included studies, they have confirmed that ligustrazine has protective effect in cerebral ischemic injury-based animal models. They have also discussed possible mechanisms behind this protective effect. Cao et al. provided a scientific metrology study to understand Traditional Chinese Medicine targeting ferroptosis. In the last 1 decade, Frontiers in Pharmacology has documented the maximum number of publications in this field. Chen et al. discussed the pharmacological and clinical potential of *Brucea javanica* (L.) Merr. and its metabolites. They have mentioned that around 200 metabolites have so far been characterized from this plant source and the most studied pharmacological application is its utility as anti-tumor agent. Clinical studies have also been recorded, which are mostly observational studies. Yu et al. explored the role of Traditional Chinese Medicine in treating and managing age-related macular degeneration and how the gut microbiota influences. This review examined the mechanistic interplay between gut microbiota dysbiosis and age-related macular degeneration (AMD), focusing on emerging evidence of bidirectional gut-retina axis signaling and its implications for AMD pathogenesis. Tang et al. focused on Armeniacae semen amarum—seeds of *Prunus armeniaca* L., and discussed its botany, phytochemistry, pharmacological and toxicological properties, pharmacokinetics, and clinical application. This natural resource holds broad spectrum therapeutic potential, including but not limited to anticancer, antidiabetic, hepatoprotective, along with many others. Li et al. focuses on the Tibetan medicine, Bang Jian, and comprehensively covered its ethnomedicinal values, phytochemistry, and therapeutic properties. Traditionally, dried flowers of this plant have been used for various lung diseases. Scientific studies validated it to have much wider pharmacological spectrum. Wu et al. discussed the extracellular vesicles obtained from traditional medicinal plants like *Zingiber officinale*, *Citrus paradisi*, *Citrus limon*, *Ginseng quinquefolium*, along with many others. It is a comprehensive review discussing various properties of such plant-derived extracellular vesicles. Susilawati et al. focused on *Erythrina* genus plants of *Fabaceae* family and discussed their pharmacological and clinical potential. This review focused on the Goal 3: Good Health and Wellbeing of the sustainable goals. Not least, Fan et al. prepared a comprehensive review which examines computational advances (network analysis, deep learning) in TCM pharmacology, analysing formula-herb-component-target-phenotype-ZHENG databases, addressing homogeneity bottlenecks, and envisioning next-gen models for multi-target TCM research.

**FIGURE 1:**
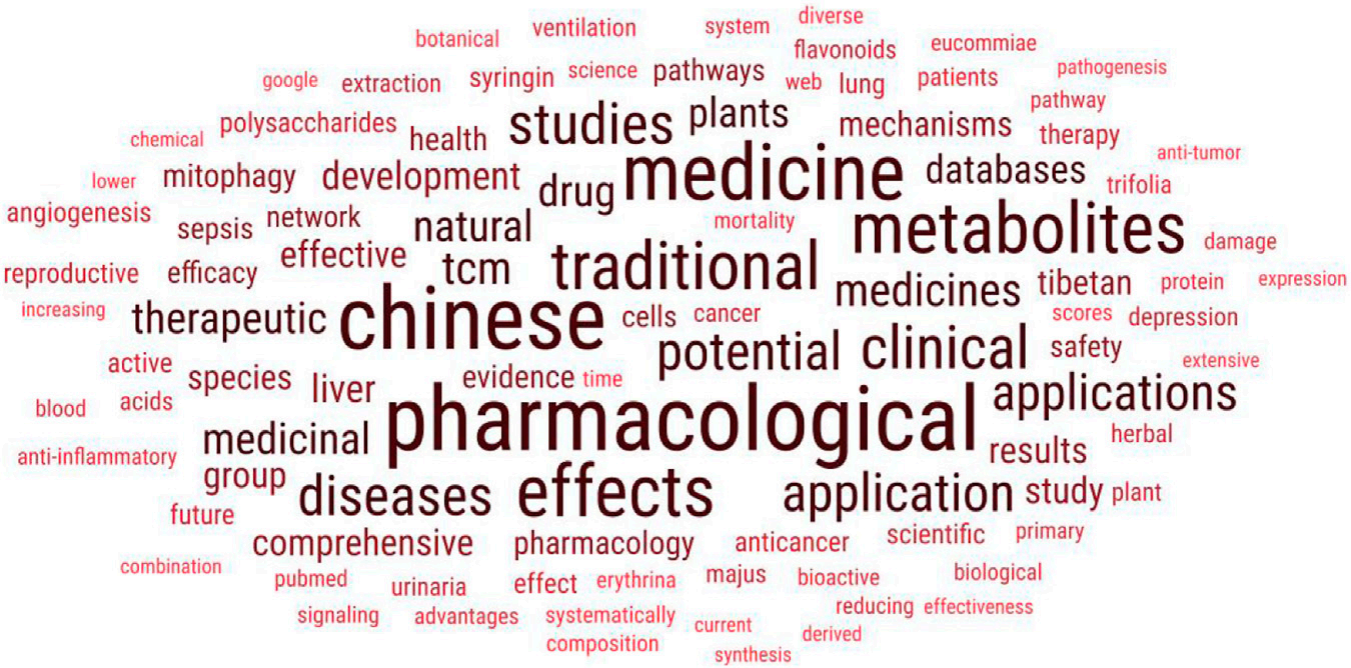
Frequency mapping of various frequently used terminologies in the abstracts of these 30 documents.

These 30 articles have definitely enriched the literature in ethnopharmacology. In order to further understand the frequently used terminologies, we have performed cloud computing on the abstracts of these 30 articles (Figure 1). Some of the most prominent keywords are Chinese, Tibetan, sepsis, anticancer, anti-inflammatory, polysaccharides, along with many others.

We are highly thankful to all the authors who have significantly contributed their high-quality articles in our Research Topic and we strongly encourage them to further promote it on social media, including X (previously Twitter). They can use some hashtags like #INPST, #DHPSP, #NPMND, #SCINATMED, #AcademicTwitter, #SCICOMM, etc., which can further help to gain broader readership to these articles, and in this way, these articles can reach to wider audience (Kletecka-Pulker et al., 2021; Singla et al., 2023a).

Considering the tremendous success of this potential Research Topic, we have also released 2^nd^ volume of this Research Topic, as “Reviews in Ethnopharmacology: 2025”. We invite the authors, readers, and reviewers to contribute in this Research Topic and submit their review articles.
